# Recent Advances in CRISPR/Cas System-Based Biosensors for the Detection of Foodborne Pathogenic Microorganisms

**DOI:** 10.3390/mi15111329

**Published:** 2024-10-30

**Authors:** Sanlei Xie, Yuehong Yue, Fan Yang

**Affiliations:** 1 College of Animal Science and Technology, Henan University of Science and Technology, Luoyang 471023, China; fyang@haust.edu.cn; 2 College of Veterinary Medicine, Southwest University, Chongqing 400715, China; yueyaohong123@126.com

**Keywords:** clustered regularly interspaced short palindromic repeats associate system (CRISPR/Cas), biosensor, foodborne pathogen, nucleic acid detection

## Abstract

Foodborne pathogens pose significant risks to food safety. Conventional biochemical detection techniques are facing a series of challenges. In recent years, with the gradual development of CRISPR (clustered regularly interspaced short palindromic repeats) technology, CRISPR/Cas system-based biosensors, a newly emerging technology, have received much attention from researchers because of their supreme flexibility, sensitivity, and specificity. While numerous CRISPR-based biosensors have a broad application in the field of environmental monitoring, food safety, and point-of-care diagnosis, they remain in high demand to summarize recent advances in CRISPR/Cas system-based biosensors for foodborne pathogen detection. In this paper, we briefly classify and discuss the working principles of CRISPR/Cas systems with trans-cleavage activity in applications for the detection of foodborne pathogenic microorganisms. We highlight the current status, the unique feature of each CRISPR system and CRISPR-based biosensing platforms, and the integration of CRISPR-Cas with other techniques, concluding with a discussion of the advantages, disadvantages, and future directions.

## 1. Introduction

Foodborne bacteria and their toxins are able to induce diseases with high mortality, posing a serious threat to human health. Foodborne pathogens contaminate raw food materials (vegetables, fruits, milk, meat, etc.) and water sources during food production, processing, storage, and transportation. Common foodborne pathogens include *Salmonella typhimurium* (*S. typhimurium*), *Staphylococcus aureus* (*S. aureus*), *Listeria monocytogenes* (*L. monocytogenes*), *Vibrio parahaemolyticus* (*V. parahaemolyticus*), *Campylobacter* spp., and *Escherichia coli* (*E. coli*) [1]. When humans directly consume or indirectly ingest contaminated food, which may be contaminated with various foodborne pathogens, these infections can lead to symptoms ranging from mild to severe state, such as diarrhea, vomiting, headache, nausea, and even death [2]. The WHO stated that about 70% of foodborne illnesses are closely related to infections caused by foodborne pathogens and viruses in food. According to a report by the CDC (Centers for Disease Control and Prevention), foodborne pathogens are responsible for causing 8 million diseases, 3000 deaths, and 128,000 hospitalizations in the United States each year [3]. Therefore, the establishment of rapid, accurate, and sensitive methods for the detection of foodborne pathogenic bacteria is of great significance to safeguard food safety and human health.

Conventional methods for detecting foodborne pathogens are based on microbiological cultures and biochemical identification, which are considered to be the “gold standard”, but currently, they are time-consuming, have low sensitivity, are labor-intensive, and have higher costs and other shortcomings, making them unsuitable for outbreaks of foodborne disease detection and analysis. In recent years, the current mainstream diagnostic methods remain molecular detection technology. Molecular-based detection technology, through the specific nucleic acids of pathogenic bacteria, designs relevant primers for nucleic acid chain amplification, including a typical amplification technique called Polymerase Chain Reaction (PCR). The combination of nucleic acid amplification with signaling tools such as agarose gel electrophoresis and quantitative real-time PCR (qPCR) allows for qualitative or relative quantification of the assay, depending on the assay requirements [4]. The fluorescent PCR method has a high sensitivity and is widely used in the detection of pathogenic bacteria. However, the non-specific amplification generated during PCR may lead to false-positive results, limiting the application of PCR [5]. ELISA based on the immune reaction between antigen and antibody has the advantages of simple operation and short detection time, but the accuracy and sensitivity are low [6]. To meet the demand for the immediate detection of foodborne pathogens, a promising technology with good specificity, higher accuracy, and sensitivity needs to be developed.

In recent years, the CRISPR/Cas system, which consists of clustered regularly interspaced short palindromic repeats (CRISPR) and associated protein (Cas), has been developed as a novel biological tool in the fields of gene editing [7], gene transcription regulation [8], and so on. In addition, the CRISPR/Cas system performs deoxyribonuclease (DNase) and ribonuclease (RNase) activities [9,10], which can be used for biosensors by directing RNA (guide RNA (gRNA)) to target nucleic acids, activating the nuclease activity of related Cas proteins, and providing a theoretical basis for the development of biosensors. Currently, the CRISPR/Cas13a-based specific high-sensitivity enzymatic reporter unlocking (SHERLOCK) [11] and the CRISPR/Cas12a-based DNA endonuclease-targeted CRISPR trans reporting system (DNA endonuclease-targeted CRISPR trans reporter, DETECTR) [10] represent a variety of nucleic acid detection technologies that have been successfully developed and applied in various fields such as medical diagnosis, life science, and food safety, and have great potential to enhance the detection of foodborne pathogenic bacteria.

In this paper, we reviewed the progress of clustered regularly interspaced short palindromic repeats (CRISPR) and CRISPR-associated protein (Cas) systems in foodborne pathogen detection, including the classification of the CRISPR/Cas system. We explained the mechanism of the CRISPR system, which involves the Cas nuclease and its role in biosensing. We summarized the existing CRISPR/Cas-based multiplexed foodborne pathogen detection methods and their research progress in recent years, analyzed the challenges faced in their practical application, and discussed the problems and challenges of these detection methods, as well as the prospects for its future development, aiming to provide new ideas for the rapid detection of foodborne pathogens.

## 2. Classification of CRISPR/Cas System

The clustered regularly interspaced short palindromic repeats (CRISPR) and CRISPR-associated protein (Cas) system is an acquired immune system found in most bacteria and archaea [12,13,14]. According to the composition of Cas proteins, the CRISPR/Cas system is mainly divided into two classes, Class1 and Class2, which can be further divided into different subtypes according to the structure and sequence of Cas proteins; Class1 is a multi-protein effector complex composed of multiple subunits, including types I, III, and IV. Class2 is a single effector protein, including Cas9 in type II, Cas12a in type V, Cas12a in type VI, Cas12a and Cas13a in type VI, etc. (Table 1). Among them, Cas9 has nucleic acid endonuclease activity with two catalytic sites (namely HNH and RuvC domains), which specifically recognizes and cleaves target double-stranded DNA (dsDNA) containing the Protospacer adjacent motif (PAM) under the guidance of transactivating crRNA (tracrRNA) [15].

Jennifer Doudna et al. integrated the two RNAs into a single guide sgRNA to make it easy to use [10,16]. Unlike Cas9, Cas12a of type V has a RuvC-like nuclease domain that specifically recognizes the target double-stranded DNA (dsDNA) and then activates non-specifically trans-cleavage activity to target other single-stranded DNA (ssDNA) or RNA [17]. Type VI Cas13 (Cas13a, Cas13b, and Cas13c) contains the HEPN domain, which has ribonuclease activity; it will non-specifically cleave the surrounding RNA guided by CRISPR RNA (crRNA). In recent years, the CRISPR/Cas system has been widely used in various fields, and the specific recognition of nucleic acid by Cas9 makes it an ideal tool for the detection of nucleic acids, and the combination of nucleic acid amplification and specific signal sensing methods, such as fluorescence, enables highly sensitive detection. The trans-cleavage activity of Cas12, Cas13, and Cas14 provides additional possibilities for the molecular diagnosis of nucleic acids, and the formation of ternary complexes stimulates nonspecific cutting activity when Cas proteins bind to target sequences under the guidance of gRNA, which can degrade any single-stranded DNA or RNA present in the system. When short-stranded DNA or RNA with fluorophores and quenching groups are added to the system, Cas proteins recognize the target sequences and cleave the reporter probes so that the fluorophore is far away from the quenching group and the fluorescence is restored [18,19,20,21,22]. The CRISPR/Cas system has been combined with a range of signaling readout modalities to design a variety of biosensors for foodborne pathogen detection.

### 2.1. Type II Effector Proteins

The Cas9 protein of the CRISPR/Cas system is the most representative type II effector protein and the first Cas protein to be applied in the field of nucleic acid detection [23]. The Cas9 protein recognizes the G-rich protospacer adjacent motif (PAM), which cleaves the dsDNA near the PAM sequence. According to the characteristics of crRNA and trans-activating crRNA (tracrRNA), we designed single guide RNA (sgRNA), which guides the Cas9 protein to identify dsDNAs with a PAM of 5′-NGG-3′ and binds to their upstream sequences through the HNH domain to cleave the complementary strand paired with the sgRNA and the other non-complementary strand through the RuvC domain [24], thereby causing dsDNA to break and produce blunt ends. In addition, researchers also obtained dCas9 (dead Cas9) with a loss of endonuclease activity by mutating the H840 site (HNH domain) and the D10 site (RuvC domain), but the dCas9 protein can still specifically recognize the target DNA under the guidance of the sgRNA [25]. Because of its highly specific binding ability, the dCas9 protein has been used in amplification-free nucleic acid detection [26,27].

### 2.2. Type V Effector Proteins

Cas12 is divided into three subtypes: Cas12a (Cpf1), Cas12b (C2c1), and Cas12c (C2c3). Of these, Cas12a is the most common. The high temperature requirement of Cas12b limits its application in the field of biosensing and detection, as its optimal DNA cleavage activity occurs at 48 °C [28], while Cas12a only needs a 37 °C temperature. Thus, Cas12a is still widely used at present. Cas12a is a single RNA-required effector protein, which requires only a single crRNA and recognizes the 5′-(T)TTN-3′ PAM sequence upstream of the target dsDNA, and at the same time, activates the trans-cleavage activity to non-specifically cleave single-stranded DNA (single-stranded DNA, ssDNA) [10]. The RNA-mediated pattern of Cas12b protein is similar to that of Cas9 protein, which is guided by both crRNA and tracrRNA and contains a single RuvC domain [29].

The Cas12f protein (formerly known as Cas14) consists of 400–700 amino acids, is half the size of other class II Cas effector proteins, has a RuvC domain, and can be guided by both crRNA and tracrRNA, which recognizes and cleaves the target ssDNA without any PAM requirement [21]. In addition to targeting ssDNA, it has PAM (5′-TTTR-3′)-dependent dsDNA cleavage activity [30]. Cas12f is capable not only of target-sequence cleavage (cis-cleavage) but also of the collateral cleavage of untargeted sequences (trans-cleavage). Cas12f has a higher specificity, as base mismatches in the target DNA can severely affect its activity [21].

### 2.3. Type VI Effector Proteins

Cas13a, a signature member of Type VI, is a class of single effector RNA-guided RNA nuclease. There are two HEPN (higher eukaryotes and prokaryotes nucleotide, HEPN) domains associated with the RNase activity of Cas13 [9,31]. Due to its lack of a RuvC domain, it has no DNase activity [32]. Guided by a gRNA with a 28 nt spacer, Cas13 recognizes target RNA containing a protospacer flanking site (PFS) and cleaves RNA via two HEPN domains. Cas13 protein does not need a PAM sequence but requires a protospacer-flanking site (PFS), and the 3′ end of PFS must be adenine, cytosine, or uracil [33]. Guanine bases affect the complementary base pairing between crRNA and target ssRNA [9]. Single base mismatches between the crRNA and the target have a very low impact on the activity of Cas13a, but two or more base mismatches can severely reduce the activity of Cas13a.

## 3. CRISPR/Cas-Based Biosensors on Foodborne Pathogen Detection

Currently, CRISPR/Cas-based biosensors have been developed to detect nucleic acid sequences of pathogens via nucleic acids or probes, which amplify biological signals and transform them into easily detectable physicochemical signals, and the purpose of qualitative or quantitative detection of pathogens can be realized through processing these physicochemical signals. According to the principle of CRISPR/Cas-based biosensors, they can be categorized into colorimetric sensors, fluorescent sensors, and electrochemical sensors (Table 2). This paper focuses on several common detection sensors based on the CRISPR/Cas system. These various CRISPR/Cas-based biosensors subsections are therefore organized according to which Cas effector proteins and signaling probe they utilize.

### 3.1. Fluorescent Biosensors Based on the CRISPR/Cas System

After some substances are irradiated by ultraviolet light, they are in an excited state, and a transition between atoms’ energy levels will occur, thus emitting a variety of visible light, making the substance fluorescent [79,80]. Based on the principle of fluorescence generation, fluorescent biosensors (FBs) enable the accurate and rapid detection of pathogens [81]. After adding a specific target, the whole system will respond to this stimulus and generate the output signal in the form of fluorescence, and the detection of the target is realized by recognizing the fluorescent signal. As the earliest biosensor used in the CRISPR/Cas system, it is also one of the most widely used methods at present. The CRISPR/Cas-based fluorescent biosensor system mainly realizes fluorescence detection in two ways: fluorescent cutting probes and fluorescent dyes.

#### 3.1.1. Fluorescent Biosensors Based on Fluorescent Cutting Probes

**Figure 1:**
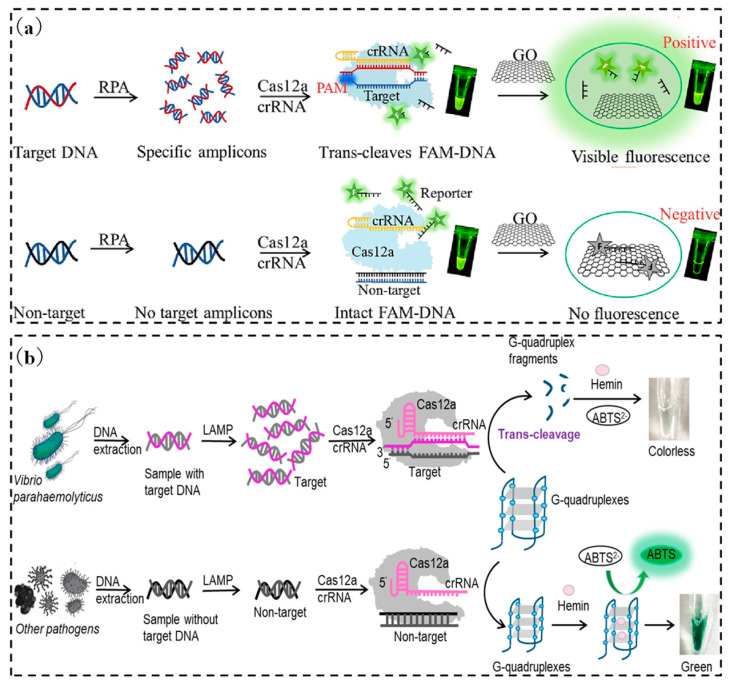
Fluorescence biosensing based on CRISPR/Cas12. (**a**) Detection method for Salmonella based on CRISPR/Cas12a and graphene oxide. (**b**) Label-free colorimetric biosensors based on CRISPR/Cas12a and G-quadruplexes.

This class of biosensors based on fluorescent cleavage probes mainly utilizes the “collateral cleavage” activity of Cas12 and Cas14 of type V and Cas13 of type VI [9,82,83]. The fluorophore quencher-labeled reporter (FQ Reporter) is used in this class of biosensors, and single-stranded DNA (ssDNA) or single-stranded RNA (ssRNA) ligated with fluorophores and quenching moieties to form ssDNA-FQ or ssRNA-FQ [11,18,21]. Under normal conditions, there is no fluorescence in the system, but the fluorophores and quench groups of ssDNA-FQ or ssRNA-FQ will be separated to restore fluorescence signals when Cas effector proteins with DNase or RNase are present in the system (Figure 1).

The principle of this fluorescent reporter is similar to that of the TaqMan fluorescent probe used in qRT-PCR. The difference is that each strand amplified in qRT-PCR produces a fluorescent molecule, while the “collateral cleavage” activity of the Cas effector protein cleaves a large number of fluorescent reporters once activated, thus producing more fluorescent molecules. For example, by modifying fluorescent and quencher groups on ssDNA, when Cas12a or Cas12b is activated in the presence of the target gene and undergoes trans-cleavage, the target gene signal can be converted into a fluorescent output signal [84,85,86]. Wang et al. [87] developed a single fluorescent motif based on the principle that graphene oxide can adsorb long ssDNA (F-ssDNA) modified by FAM and quench its fluorescence (Figure 1a). The trans-cleavage activity of Cas12a shortens the length of DNA F-ssDNA, resulting in the reduced adsorption capacity of graphene oxide and poor fluorescence quenching effect. Compared with double-labeled ssDNA, this method can save 65% of the cost. The detection limit was as low as 50 copies when *Salmonella* was used as the detection target.

The CRISPR/Cas12 system coupled with isothermal amplification utilizes the trans-cleavage activity of Cas12 to detect target ssDNA. This is combined with fluorescence signal or immunochromatographic test strips, which are widely used in the field of foodborne pathogen detection [88,89,90,91,92,93]. In addition to the trans-cutting activity of ssDNA, Cas12a can also cut DNA with advanced structures, such as G-quadruplex DNA [94]. Wang et al. [43] utilized G-quadruplex to enhance the fluorescence emission of specific ligands, whereas the trans-cleavage of Cas12a disrupted the advanced structure of G-quadruplex, thus failing to enhance the emission signal of the fluorescent ligands. From this, we constructed a labeling-free fluorescence detection method, which has a limit of detection for *Vibrio parahaemolyticus* as low as 136 copies. In addition, the complex of G-quadruplex DNA bound to heme has peroxidase-like activity, which can catalyze the oxidation reaction of the chromogenic substrate and produce color change. This catalytic activity and color change cannot occur when the G-quadruplex structure is disrupted by the trans cleavage of Cas12a (Figure 1b) [63]. The limit of detection of this method is 9.8 CFU per reaction for *Vibrio parahaemolyticus*. Due to the targeted recognition of DNA by Cas12, it can be combined with most nucleic acid amplification methods, and its trans-cutting activity can further improve the sensitivity and realize flexible signal conversion and output [44]. Liu et al. [95] combined Cas12a with RPA for the detection of *Escherichia coli*, *Listeria monocytogenes*, *Staphylococcus aureus,* and *Vibrio parahaemolyticus*. It was also found that the RPA-Cas12a two-step assay was able to increase the sensitivity by more than 10-fold compared to the traditional RPA-conjugated lateral flow test strips and identified DNA at the 10-copy level [44]. An et al. [96] developed the RPA-Cas13a method for the *Salmonella*-specific invA gene and validated both two-step and one-step assay systems. An also pointed out that the RPA-Cas13a one-step method is convenient and fast while the detection limit reaches the level of traditional PCR; the two-step RPA-Cas13a method has a more complicated process, but the detection limit is significantly reduced by more than 10-fold. Li et al. [97] developed a two-step RPA-Cas12a assay for the detection of the fimY gene of *Salmonella* and successfully applied it to the detection of *Salmonella* in eggs. Zhu et al. [46] combined Recombinase Aided Amplification (RAA) technology similar to RPA with Cas12a to establish fluorescence and test strip method for *E. coli O157:H7*. The sensitivity is higher than that of traditional real-time fluorescent PCR and ELISA methods. Tian et al. [47] established an ultrasensitive RPA-Cas12a reaction platform for *Listeria monocytogenes* and achieved isolation from the outside environment through a fixed addition container, which not only greatly simplified the operation process but also exceeded the sensitivity of the one-tube method. Xia et al. [48] combined RPA and Cas12a in the same container to detect *Listeria monocytogenes*, and the limit of detection was 4.4 CFU/g in spiked pork within 25 min. Zhou et al. [49] combined RAA and Cas12a to establish a test strip assay for *Staphylococcus aureus*, and the detection limit was as low as 5.4 × 10^2^ CFU/mL. Lv et al. [50] combined RAA with Cas12a for the detection of *Vibrio parahaemolyticus* and demonstrated that the addition of bovine serum proteins and proline could enhance the reaction.

Traditional LAMP technology typically employs the binding of fluorescent dyes to double-stranded DNA, which is prone to causing non-specific binding and leading to false positives [98]. Amplification of target DNA fragments using LAMP not only increases the sensitivity of CRISPR-based detection technology but also effectively alleviates the false positives problem of LAMP. Lee et al. [52] combined LAMP and Cas12a with the addition of reporter DNA modified with FAM and biotin (Biotin) at both ends to detect *Salmonella*. In the detection system, if the target gene is absent, the non-specific cleavage property of Cas12a will not be activated, and both the T-line for detecting the FAM–biotin complex and the C-line in the test strip will exhibit color. When the target gene fragment is present, the reporter molecule undergoes cleavage, and the T line fails to show color; only the C line can show color. Additionally, Lee et al. [99] also established a LAMP-Cas12a fluorescence detection method for *E. coli O157:H7*, which consisted of 20 min of DNA release, 40 min of LAMP amplification, and 5 min of Cas12a fluorescence color manifestation. Li et al. [93] developed a method using immunocapture magnetic beads (MBs) to enhance the sensitivity of CRISPR/Cas12a-LAMP for the rapid visual detection of *Campylobacter jejuni*. The method completes the detection within 8 min with the LOD as low as 70 CFU/mL. The results indicated that the combination of immunocapture MBs, LAMP, and CRISPR/Cas12a system could significantly enhance the sensitivity and specificity of detection of *Campylobacter jejuni*. Hu et al. [53] integrated LAMP and Cas12a and established a LAMP-Cas12a detection approach for *Vibrio paraholyticus* by utilizing self-screened target genes. By adding the TTTT sequences to the LAMP primers, Hu successfully addressed the issue of the absence of PAM binding sites in the self-screening primers. Shi et al. [54] combined Cas12a with LAMP to develop a two-step rapid fluorescent detection method for *Shigella flexneri* that can be visually observed by a blue LED light, featuring high sensitivity and specificity.

**Figure 2:**
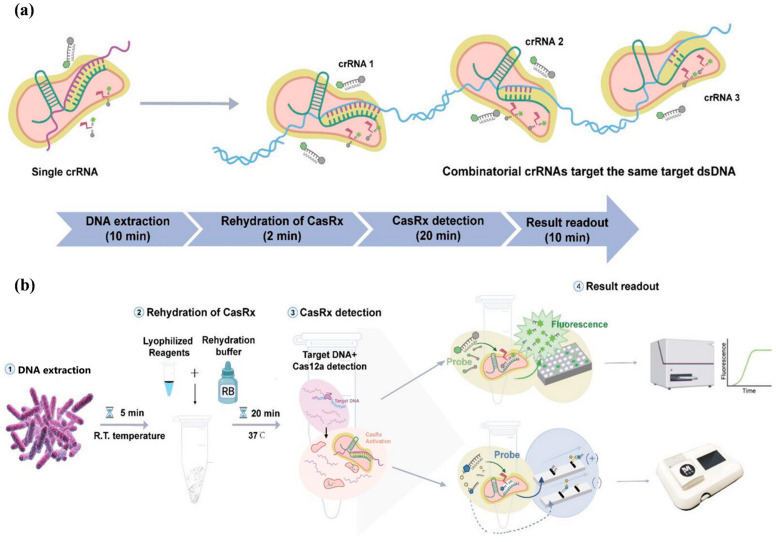
Schematic of amplification-free detection of drug-resistant Salmonella. (**a**) Schematic of comparing the multiplex-crRNA strategy with the single-crRNA method in the CRISPR/Cas12a system; (**b**) schematic diagram of the AFC-TRFIA workflow, including the sample genomic DNA extraction, sample addition, CRISPR/Cas12a reaction, and resultant readout.

Bao et al. [55] developed an amplification-free CRISPR-Cas12a time-resolved fluorescence immunochromatographic assay based on the design of a dual-crRNA system for the detection of *Salmonella* with a 27-fold increase in LOD (Figure 2). The method designs ssDNA-1 and ssDNA-2 based on the principle of complementary base pairing and couples Eu(III)-time-resolved fluorescent microspheres (TRFM) with an anti-biotin antibody, and binds to biotin-modified ssDNA-1 to form an alternative signaling reporter probe (TRFM- biotin-ssDNA-1) that replaces traditional ssDNA-FQ. When the target is present, Cas12a collaterally cleaves the signaling reporter probe ssDNA-1 so that the T-line-coated ssDNA-2 probe designed to recognize TRFM-biotin-ssDNA cannot bind to it, resulting in no fluorescence on the T line. Meanwhile, the C-line aggregates and produces obvious fluorescence due to the anti-biotin antibody being captured by the secondary antibody. When the target is not present, the TRFM-biotin-ssDNA-1 reporter probe cannot be cleaved by Cas12a and is captured by the ssDNA-2 probe on the T-line, and the antibody is also captured by the secondary antibody on the C-line, and then both the T-line and the C-line emit visible fluorescence under UV irradiation at the same time, and the platform can detect drug-resistant *Salmonella* in 30 min with the linear range of detection from 4.9 × 10^2^~1.6 × 10^6^ CFU/mL, and the calculated limit of detection reaches 84 CFU/mL. Using a similar principle, Zhou et al. [49] established a CRISPR/Cas-recombinase-assisted amplification lateral flow analysis sensor (CRA-LFB) for *S. aureus* detection based on streptavidin-modified quantum dots and recombinase-aided amplification (RAA). This LFB sensor has a detection limit of 75 amol/L for *S. aureus* genomic DNA and 5.4 × 10^2^ CFU/mL for a bacteria solution within 70 min. Compared with colloidal gold particles and fluorescent dyes, quantum dot labeling has high fluorescence characteristics and good stability [100], as well as the advantages of higher sensitivity and lower background interference on LFB strips [101].

Gootenberg et al. [11] developed the Specific high-sensitivity enzymatic reporter unlocking (SHERLOCK) platform, which utilizes Cas13a to recognize the RPA product and trans-cut the reporter to release the fluorescent signal; this method can be used to detect target bacteria such as the *Zika virus Dengue virus*, *Escherichia coli*, and other target bacteria in 1 h. The detection limit was as low as 20 amol/L for the *Zika virus*.

#### 3.1.2. Fluorescent Biosensors Based on Fluorescent Dyes

Fluorescent dye-based biosensors mainly utilize the CRISPR/Cas system to identify the target, which uses fluorescent dyes to label the amplicon and achieve target detection with the help of real-time fluorescent PCR and other tools.

Huang et al. [102] developed an efficient nucleic acid detection system (Cas-EXPAR) by combining the cleavage effect of CRISPR/Cas9 with the efficient amplification effect of exponential amplification reaction (EXPAR). When there is a PAM-presenting oligonucleotide (PAMmer) in the system, the target ssDNA or RNA is cleaved at a specific site under the action of Cas9/sgRNA, and the short oligonucleotide fragment obtained after cleavage acts as a primer for the EXPAR. The method generates a large number of DNA amplicons after a period of time, and then the amplified DNA can be detected by real-time fluorescence monitoring, which converts the amplified amplicons into the form of fluorescent signals. With the strong amplification ability of EXPAR, the detection sensitivity of Cas-EXPAR can be increased to 0.82 amol/L, and it is able to distinguish the single base mismatch at the cleavage site. In addition, Cas-EXPAR enabled the detection of DNA methylation and *Listeria monocytogenes* mRNA. Compared with traditional nucleic acid amplification, this system does not require the addition of exogenous primers, which avoids the non-specific amplification in the conventional amplification reaction and ensures the efficiency of amplification. However, due to the excessive detection steps and the required reagents being too complicated, the further application of Cas-EXPAR is hindered.

**Figure 3:**
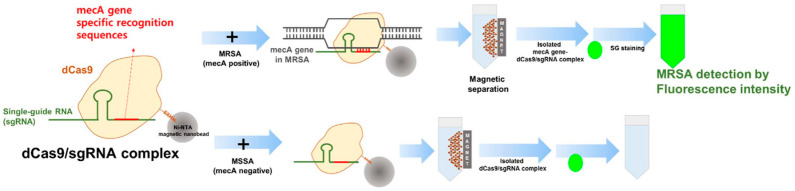
Fluorescent biosensors based on dCas9/sgRNA-SG DNA-FISH fluorescent dye.

With the help of SYBR GREEN I fluorescent dye, Guk et al. [38] developed a dCas9/sgRNA-SG I-based DNA-FISH assay that can selectively detect *methicillin-resistant Staphylococcus aureus (MRSA)* strains. In this system, the sgRNA sequence is designed to recognize the mecA resistance gene, and the CRISPR-associated protein 9/single-guide RNA (dCas9/sgRNA) complex specifically recognizes the mecA gene and forms a ternary complex upon addition of the target. SYBR green I (SG) is a well-known sensitive fluorescent dye used for target DNA visualization. Unlike conventional gene diagnosis methods in which DNA extraction, isolation, and purification are needed, the target gene can be detected within 30 min with high sensitivity without performing a gene separation step by using cell lysates, and the minimum detection concentration of the assay can be up to 10 CFU/mL (Figure 3). The greatest advantage of this system is that there is no additional amplification means, and the target nucleic acid is enriched by dCas9/sgRNA so as to shorten detection time and reduce labor relative to that of conventional methods. The difference between Cas-EXPAR and dCas9/sgRNA-SG-based I DNA-FISH is that the Cas effector proteins are used in the two systems. The former utilizes the binding and cleavage functions of Cas9/sgRNA on the target nucleic acid, while the dCas9/sgRNA complex has advantages over monoclonal antibody, which is able to bind the target DNA in a sequence-specific manner but does not cleave the target.

Wang et al. [36] developed a one-pot RNA assay for the detection of *Salmonella typhimurium* by combining Cas9 nickase and isothermal amplification technology. A detection limit as low as 0.99 pmol/L *Salmonella typhimurium* genomic DNA was detected in a 20 µL reaction system, and the system was shown to have an excellent nucleotide-mutation discrimination capability in detecting target sequences.

Sun et al. [34] developed a CRISPR/Cas9-triggered strand displacement amplification and rolling circle amplification (SDA-RCA) two-step isothermal amplification for the fluorescence detection of *E. coli O157:H7* based on the UiO9 platform. The target virulence gene sequences were recognized and cleaved by the CRISPR-Cas9 system and triggered the rolling circle amplification of SDA and RCA. After the reaction, a large number of products could be hybridized to the probe and the target DNA was quantified by fluorescence intensity. This method achieved specific detection of *E. coli O157:H7* in food matrices under mild conditions, with a minimum LOD of 40 CFU/mL and a linear range of 1.3 × 10^2^ to 6.5 × 10^4^ CFU/mL, which presented a higher reaction efficiency compared with the traditional RCA and SDA methods. The detection limit was three orders of magnitude lower than that of the RT-PCR Kit, and it also showed great potential for further application ability in other pathogenic bacteria detection and food safety detection.

### 3.2. Colorimetric Biosensors Based on the CRISPR/Cas System

Colorimetric biosensors based on the CRISPR/Cas system are a class of biosensors that use visual colors generated by the selective absorption of light in solution or rely on spectroscopic instrumentation [103]. CRISPR/Cas-based biosensors use optical characteristics for rapid and efficient pathogen detection. Unlike fluorescent biosensors, which require additional tools to read fluorescence intensity, biosensors based on colorimetric analysis methods can be visualized with the naked eye.

Single-stranded DNA with modified reporter molecules at both ends is more expensive for CRISPR-Cas fluorescence and test strip assays. Therefore, researchers have developed several signal transduction methods to activate Cas protein nuclease activity. Gold nanoparticles (AuNPs) have special optical properties and chemical binding activity, and their binding to single-stranded DNA causes an aggregation effect that can produce a visually detectable color change. When Cas proteins with DNA trans-cutting activity are added to the AuNP crosslinking system, the aggregation reaction is inhibited, and visual color change cannot be generated in the system.

**Figure 4:**
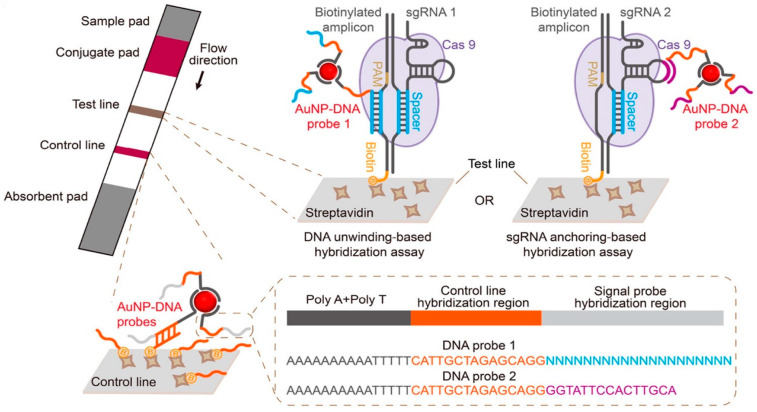
The developed CASLFA colorimetric biosensors based on the CRISPR/Cas system.

Wang et al. [37] established a novel colorimetric biosensing CASLFA (CRISPR/Cas9-mediated lateral flow nucleic acid assay) by integrating Cas9-mediated detection technology onto lateral flow chromatography (LFD) test strips. In this system, target DNA is amplified using biotinylated primers, and Cas9/sgRNA specifically recognizes the target DNA and forms a ternary complex after the amplification product is added. The resulting Cas9/sgRNA-biotinylated amplicons are then trickled onto the sample pad of the test strip. Under capillary force, the complex flows laterally through the binding pad, and the AuNP-DNA probes bound to the Cas9/sgRNA-biotinylated amplicons via nucleic acid hybridization, forming a ternary complex which is then captured by the streptavidin immobilized on the detection line, and the excess AuNP-DNA will continue to flow through the lateral flow device and be trapped at the control line by hybridizing with the capture probe. Color bands will be generated on the detection line and the control line due to the accumulation of AuNPs, and the presence or absence of the DNA target can be determined by the color display (Figure 4). The CASLFA assay was evaluated using *Listeria monocytogenes*, a transgenic 35S promoter and *African swine fever virus* (*ASFV*) at a detection limit of 150–200 copies/reaction of genome samples with high specificity within 1 h; it could be used for the detection of a wide range of foodborne pathogens. CASLFA satisfies some of the characteristics of a next-generation molecular diagnostics tool due to its rapidity and accuracy, allowing for point-of-care use without the need for technical expertise and complex ancillary equipment.

Bengtson et al. [41] proposed a CRISPR/dCas9-mediated isothermal amplification DNA detection strategy. Recombinase polymerase amplification (RPA) was performed using biotinylated primers after DNA extraction from blood/urine. dCas9/sgRNA bound to the RPA product and was immobilized by streptavidin-labeled magnetic beads. The dCas9 protein was labeled with ssDNA that hybridized to circular primers, and rolled circle amplification (RCA) was performed under the action of ligase and polymerase. The RCA product folds to form a G-quadruplex that binds to the heme moiety and catalyzes the chromogenic reaction. The method requires no complicated equipment, can be performed at room temperature, and can detect as low as 10 copies of DNA in 15 min.

Wang et al. [104] introduced PAM-mer sequence “NGG” to enable Cas9 to cut target ssRNA/ssDNA, thereby releasing specific fragments and triggering exponential amplification to generate a large number of G-rich amplification products, and realized visual detection by utilizing the characteristics of peroxidase activity after binding G-quadruplex to heme. The method targeted ssDNA fragments of *Listeria monocytogenes*, and the detection limit was as low as 100 amol/L.

**Figure 5:**
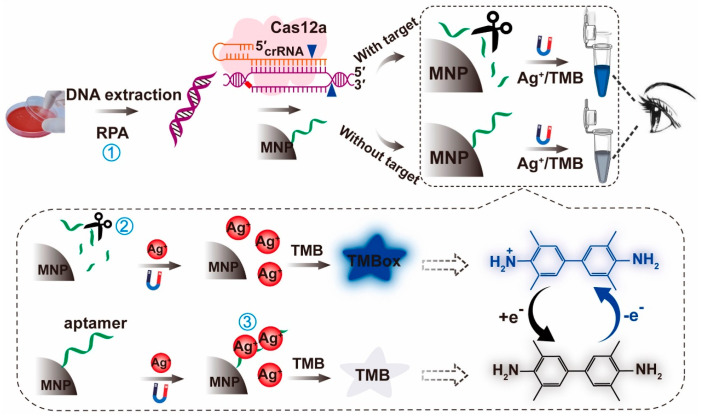
Schematic of aptamer-based colorimetric biosensor using a CRISPR/Cas12a system and RPA. ① RPA; ② CRISPR/Cas12a amplification; ③ Aptamer-Ag^+^ amplification.

Wei et al. [61] developed an aptamer colorimetric biosensor based on CRISPR/Cas12a and RPA, as shown in Figure 5, which can be used for ultrasensitive visual detection. When Ag aptamers containing large amounts of cytosine residues are present, the original free Ag in solution is reduced due to the aptamer binding, accordingly, resulting in a decrease in the amount of free Ag interaction with 3,3′,5,5′-tetramethylbenzidine (3,3′,5,5′-tetramethylbenzidine (TMB). Using this principle, the Ag-TMB chromogenic response can be modulated with Ag aptamers. This biosensor has satisfactory accuracy and applicability with LOD as low as 8 CFU/mL, which is more suitable for bacterial identification and typing for ultrasensitive and rapid visual detection. G-quadruplex, an advanced structure formed by the cation-induced folding of tandem-repeat guanine-rich DNA or RNA, possesses peroxidase-like activity upon binding to heme chloride. It can catalyze the chromogenic reaction of TMB and 2,2′-diazo-bis -(3-ethylbenzothiazoline 6-sulfonic acid) (2,2′-azino-bis-(3-ethylbenzothiazoline 6-sulfonic acid) (ABTS), often as an output signal of colorimetric mode. Based on this principle, researchers have developed a corresponding CRISPR/Cas-isothermal amplification colorimetric sensing platform.

**Figure 6:**
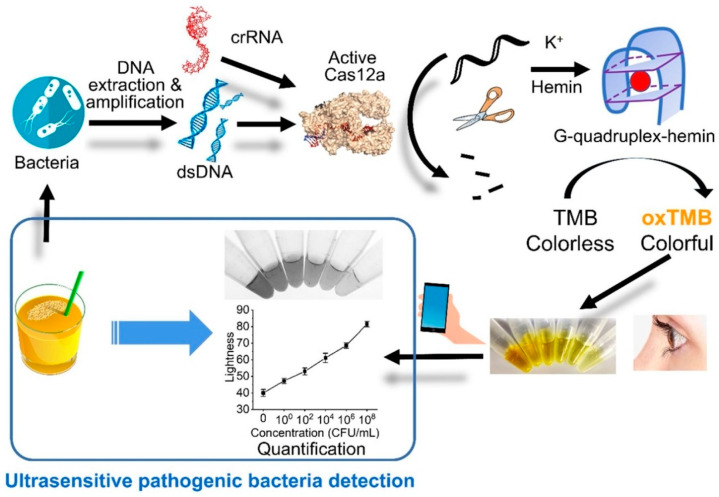
Ultrasensitive *Salmonella*-specific invA gene detection by a smartphone-read G-quadruplex-based CRISPR-Cas12a bioassay.

Yin et al. [62] cleverly designed a smartphone-based CRISPR/Cas12-RPA bioassay for the ultrasensitive detection of bacteria. As shown in Figure 6, the amplicon triggers the degradation of ssDNA by the Cas12 effector protein in the presence of the target substance and cannot catalyze TMB chromogenic reaction. When the target substance is not present, guanine-rich ssDNA forms a stable G-quadruplex DNase by the addition of K^+^, which catalyzes a color change in the TMB-H_2_O_2_ reaction in the presence of heme and can be directly observed by the naked eye or a smartphone with a color picker. Applying this strategy, the LOD of *Salmonella* was 1 CFU/mL. This technology expands the application of the CRISPR/Cas biosensing system and provides a novel detection platform with high sensitivity and specificity for pathogenic bacteria in food, which can be used as an effective food safety assessment tool for foodborne pathogens. Based on the same principle, Chen et al. [63] developed a CRISPR/Cas12a combined with G-quadruplex DNAzyme visual detection platform to detect *Vibrio parahaemolyticus* in food. CRISPR/Cas12a-LAMP induces the chromogenic development of the G-quadruplex DNase-catalyzed substrate ABTS with a sensitivity of 6.1 × 10^2^ CFU/g for naked-eye detection. This cascade approach can serve as a universal biosensing strategy for on-site pathogen detection.

In addition, in order to detect multiple foodborne pathogens simultaneously, Wang et al. [35] developed a dual detection method for foodborne pathogens by combining CRISPR/Cas9n and test strips, in which Cas9n is obtained by site-directed mutation of the Cas9 protein, and only the HNH active domain is retained [105]. The authors first used Cas9n protein to establish a thermostatic duplex DNA amplification technique against *E. coli* and *Salmonella typhimurium*, followed by labeling digoxin and biotin on both sides of the *E. coli* amplicon and FITC and biotin on both sides of the *Salmonella typhimurium* amplicon, and encapsulating anti-digoxin antibody in the T1 line of the strip, anti-FITC antibody in the T2 line, and biotinylated secondary antibody in the C line for binding excess streptavidin-labeled colored latex microspheres. The method could detect two foodborne pathogens at the same time, and the detection limit for genomic DNA was only 100 copies/μL, and the detection limit for bacterial solution was 10^2^ CFU/mL. In addition, Wang et al. [40] also designed a test strip for the detection of *Salmonella* using the Cas9 protein, which can detect a minimum of 10^2^ CFU/mL *Salmonella* and can accurately detect contaminated milk samples.

**Figure 7:**
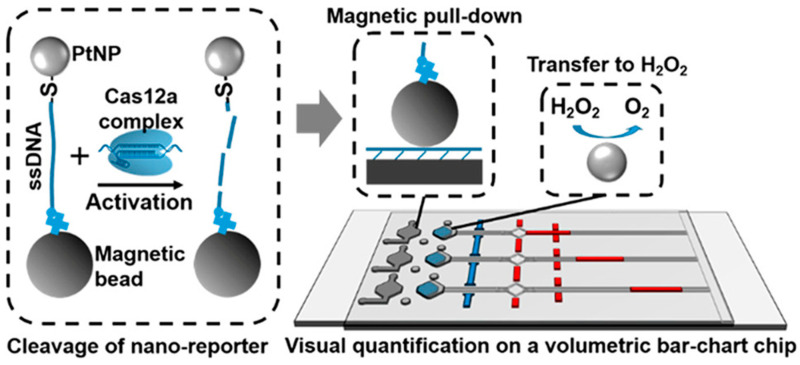
The platinum nanoreporter-based CRISPR-Cas12a detection system on the MAV-chip.

Ning et al. [106] developed a magnet-assisted V-chip (MAV-chip) based on the previously innovated Volumetric bar-chart chip (V-chip) detection platform, which combined metal platinum reporters and CRISPR-Cas12a to realize the visual and quantitative detection of pathogen nucleic acids. In this MAV-chip detection system, magnetic bead-single-stranded DNA-PtNP (BDNP) conjugated compounds were added as reporters to the CRISPR-Cas12a system. Once the Cas12a/crRNA recognizes the target DNA and excites the activity of Cas12a’s non-specific cleavage of ssDNA, the ssDNA linker in the BDNPs is degraded, thereby releasing the metallic platinum from the beads, which catalyzes the production of oxygen by H_2_O_2_, and the gas pushes the red ink forward to visualize and quantify the target (Figure 7). Compared to fluorescent, colorimetric, and electrochemical biosensors, which require specific instruments for quantitative detection, this new platinum nanoprobe-based MAV-chip does not require additional instrumentation and can be quantitatively detected by the naked eye, which greatly reduces experimental costs and improves detection efficiency.

Gold nanorods (GNRs) with different aspect ratios have unique transverse surface plasmon resonance (TSPR) and longitudinal surface plasmon resonance (LSPR) modes that exhibit different colors. These characteristics enable GNR to have good color rendering performance in chromogenic reactions. Xu’s group used the convertase-glucose oxidase cascade reaction to cause GNR discoloration and Fenton reaction readout signals [107]. The target DNA activates the trans-cleavage activity of the Cas12a system under the guidance of crRNA, which cuts off the ssDNA between the functional magnetic beads and the convertase, and the invertase released in the supernatant after magnetic separation undergoes a cascade reaction to catalyze the hydrolysis of sucrose and the resulting glucose is immediately oxidized by GOx to form H_2_O_2_. In the acidic environment, H_2_O_2_ is rapidly converted into an active intermediate (·OH) by Fe^2+^-induced Fenton reaction. The -OH etches the GNR mainly along the longitudinal axis, resulting in a significant change in the aspect ratio, thus altering the solution color. The final color can therefore be effectively correlated with the concentration of the target DNA and can be easily distinguished by the naked eye. Furthermore, the peak wavelength of the LSPR can be used for semi-quantitative detection of target DNA by UV-Vis spectrophotometry, and the sensitivity of the target DNA after recombinase-aided amplification is 1 amol/L, which is comparable to the sensitivity of a fluorescent reporter probe.

Labeling ssRNA or ssDNA on the surface of AuNPs can increase the repulsive force between particles, which is beneficial for the stable existence of AuNPs in solution. Zhang et al. [108] used thiolated ssDNA labeled on the surface of AuNPs as a substrate for Cas12a cleavage. When non-target nucleic acid activates Cas12a, the AuNPs are well dispersed by the surface-modified thiolated ssDNA, giving the solution a red color. The target nucleic acid-activated Cas12a hydrolyzed the ssDNA on the surface of AuNPs, and the repulsion between the AuNPs decreased and then aggregated, and the solution changed from red to purple. This method realizes the visual detection based on AuNPs, but its high background value and ssDNA cleavage efficiency will affect the aggregation of AuNPs, and the difference between positive and negative results is small, which limits the analytical sensitivity of this method.

The integration of horseradish peroxidase-catalyzed chromogenic reaction with the CRISPR-Cas system is broadly divided into two categories: direct and competitive methods. Wu et al. [64] achieved a visual detection of methicillin-resistant *Staphylococcus aureus* nucleic acid by a direct method, which was activated by the target nucleic acid through the activation of Cas12a to cleave the ssDNA coupled to the surface of the beads at one end and horseradish peroxidase at the other end. The horseradish peroxidase was detached from the surface of the magnetic beads and released into the solution to react with glucose, 4-aminoantipyrine and N-ethyl-N-(2-hydroxy-3-sulfopropyl)-3-methylaniline, thus realizing the visualization of the assay.

Yuan et al. [109] proposed a better detection scheme. In this protocol, the target sequence is used to activate the Cas protein to cleave the ssDNA or ssRNA linker that can hybridize the surface probe of AuNPs. In the absence of a target sequence, AuNP-DNA probes were hybridized at both ends of the linker to form an AuNP aggregation state. Secondly, this protocol uses low-speed centrifugation to settle the paired AuNPs to the bottom of the tube to reduce the interference of background color. This scheme not only realizes the orderly aggregation of AuNPs but also enhances the ratio of negative to positive results, which can significantly improve the sensitivity of the assay. However, this scheme has strict requirements on centrifugal force and time, and it is difficult to achieve point-of-care testing (POCT). Therefore, some studies have proposed a signal amplification method based on the magnetic bead method for POCT. Hu et al. [39] established a visual detection method based on CRISPR-Cas12a for *Staphylococcus aureus* based on this principle. In this method, the two-hybrid probe of AuNP-DNA at both ends was replaced by one end coupled to the surface of the magnetic bead by biotin–avidin, and the other end was hybridized with AuNP-DNA. The hybridizing probe was still trans-cleavage by Cas effector protein, and the AuNP-magnetic bead/magnetic bead was separated by a magnetic field. The content of AuNPs in the solution was observed to determine whether there was a target nucleic acid in the sample.

**Figure 8:**
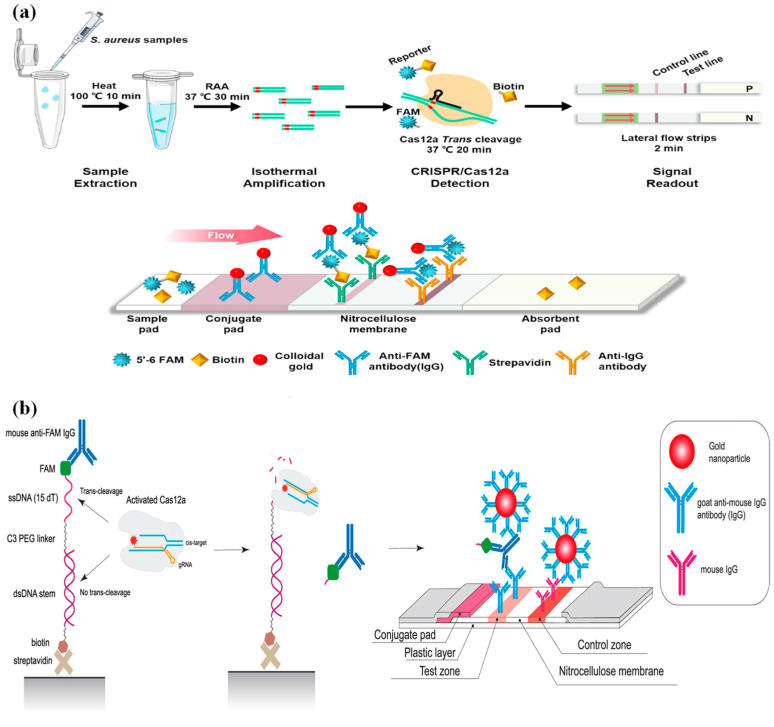
Principle of the CRISPR-based lateral flow strip assay. (**a**) CRISPR/Cas12a-based lateral flow platform for sensitive detection of Staphylococcus aureus. (**b**) DIRECT2 platform for a CRISPR-Cas12-based assay comprising a universal DNA-IgG probe and a direct lateral flow test.

Qian et al. [65] developed a method for the detection of *Staphylococcus aureus* that combines test strips and a CRISPR/Cas12a system. First, the target fragment of *Staphylococcus aureus* was amplified using RPA and then detected by the CRISPR/Cas12a system; the diluted test solution was added to the test strip and incubated to read the signal. The control line (C line) of the test strip is located at the front of the test line (T line), and the sample solution flows first through the C-line and then the T-line. The detection principle is shown in Figure 8a: the FAM-ssDNA-biotin signal reporter probe can bind to the gold-labeled anti-FAM antibody to form a complex on the binding pad. When the target is not present, the streptavidin on the C line can capture the complex through the interaction between streptavidin and biotin, and the C line shows a red color due to aggregation of colloidal gold particles, while there is no color change on the T line. When the target is present, the FAM-ssDNA-biotin signal probe is cleaved by the Cas12a protein and cut into two parts, FAM-ssDNA and ssDNA-biotin. At this time, the streptavidin on the C line binds to the ssDNA-biotin, while the FAM-ssDNA binds to the anti-FAM antibody and is captured by the anti-IgG antibody on the T line, resulting in a red color change in the T line. The more targets present, the darker the T line color, indicating the more the signal probe is cleaved, while it is the opposite for the C line. The method detected *Staphylococcus aureus* at 1 CFU/mL within 60 min, 20 CFU/mL for contaminated milk samples, and 1 × 10^2^ CFU/mL for other common food samples. In addition, Liu et al. [66] also established highly sensitive and portable CRISPR-LFA test strips for the detection of *Staphylococcus aureus* based on the above principles, which can detect as few as five copies (20 μL) of *Staphylococcus aureus* within 40 min.

The above CRISPR/Cas system-based lateral flow chromatography assays can be divided into two main categories: test strips based on the principle of biotin–streptavidin recognition and the complementary pairing principle of two ssDNA probes. The detection results of these test strips depend not only on the cleavage efficiency of the signal reporter probe but also on other factors, such as the interactions between the uncutted signal reporter probe, labeling materials (e.g., colloidal gold complexes), and receptors on the control line, the interaction between the cleaved signal reporter probe and the labeled material, and the interaction between the labeled material and receptors on the test line, and all these factors have the potential to cause false-positive results. Therefore, Ivanov et al. [110] developed a universal DNA-IgG signal reporter probe and a novel test strip based on the CRISPR/Cas system (Figure 8b) so that the color change in the test line is directly correlated with the amount of the target analyte. The authors first designed a DNA complex composed of “biotin-DSDNA-SSDNA-FAM” and combined it with anti-FAM antibodies to form a universal DNA-IgG probe, which was packaged in a 96-well plate by streptavidin. When the target is present, the accessory cleavage activity of Cas12a is activated, the ssDNA portion of the composite probe can be cleaved by the reaction solution of the 96-well plate, and then the anti-FAM antibody is released in solution, and the T-line of the test strip and the goat anti-mouse antibody on the surface of the colloidal gold particles compete for binding to a different site of the anti-FAM antibody, respectively. The platform has two test methods: long-term (90 min) and short-term (30 min), where the detection limit of the target dsDNA is 0.5 nmol/L for the long-term program and 1 nmol/L for the short-term program. The method can complete the detection of different targets by changing the crRNA.

### 3.3. Electrochemical Biosensors Based on the CRISPR/Cas System

Electrochemical biosensors are a class of biosensors that use the components of the organism or the organism itself as the sensing element, the electrode as the conversion element, and the potential or current as the signal output [111,112]. In recent years, electrochemical biosensors based on CRISPR/Cas systems have attracted much attention due to their high sensitivity, short detection time, and portability of instruments. This type of sensor mainly converts the genomic information of the pathogen into an electrical signal and can realize the quantitative detection of the target by establishing the relationship between the electrical signal and the target concentration.

Suea-Ngam et al. [67] presented an amplification-free electrochemical CRISPR/Cas biosensor utilizing silver metallization (termed E-Si-CRISPR). The electrode surface is modified with ssDNA and CRISPR/Cas12a, and when a target gene is present, the trans-cleavage activity of Cas12a is activated, and the ssDNA on the surface of the electrode is degraded. Using a custom-designed guide RNA (gRNA) targeting the mecA gene of MRSA, the Cas12a enzyme allows highly sensitive and specific detection when employed with silver metallization and square wave voltammetry (SWV). The MRSA gene was used as the detection object, and the detection limit was 3.5 fmol/L, and the quantification limit was 10 fmol/L.

Huang et al. [68] constructed a molecular reporters electrode with sulfhydryl-ssDNA-immunomagnetic beads as the main structure, and when activated Cas12a was added to the electrode, the sulfhydryl group cleavage led to a decrease in the current signal, and positive signal transduction was achieved. Huang et al. used *Salmonella* and *Staphylococcus aureus* as targets to construct a cascade assay, including Saltatory Rolling Circle Amplification (SRCA)-Cas12a-molecular electrode, with a detection limit of 3 CFU/mL.

Chen et al. [69] designed an ultrasensitive and specific electrochemical biosensor for *E. coli O157:H7* based on the combination of immune RCA and CRISPR/Cas12a. After the target *E. coli O157:H7* was introduced into the biosensor, its electrochemical signal was changed. Under the optimized conditions, the linear range of the biosensor can reach 10–10^7^ CFU/mL, and the limit of detection (LOD) is as low as 10 CFU/mL, which is significantly better than the previously reported method. Thus, a novel ultra-sensitive and specific biosensor platform for the detection of pathogens can be developed by combining Cas12a and immune RCA, which has demonstrated excellent performance for the detection of many pathogenic bacteria in real samples.

Liu et al. [70] developed a strand displacement amplification (SDA)-assisted CRISPR/Cas12a electrochemiluminescence (ECL) biosensors for ultrasensitive identification of *Staphylococcus aureus*. In this study, porphyrin Zr metal–organic framework (MOF) nanomaterials were prepared as co-reactant accelerators to enhance the reaction with QDs and amplify the ECL emission signal. The platform exhibited desirable assay performance with a linear range of 1 fmol/L to 10 nmol/L and a detection limit of 0.437 fmol/L. This CRISPR/Cas-isothermal amplification detection system on the foundation of substantial single-stranded DNA products (SP) as the signal output and the design of different crRNAs can be used to detect different pathogens, which has the advantages of low cost and fast speed.

Bu et al. [71] designed a functional DNA aptamers locked by the hairpin of primer exchange reaction (PER), and the target pathogen could bind to the aptamer and expose primer-complementary regions in the hairpin structure, triggering an amplification reaction to produce Cas12a-activating ssDNA, which was subsequently detected by an electrochemical biosensing platform mediated by Cas12a.

**Figure 9:**
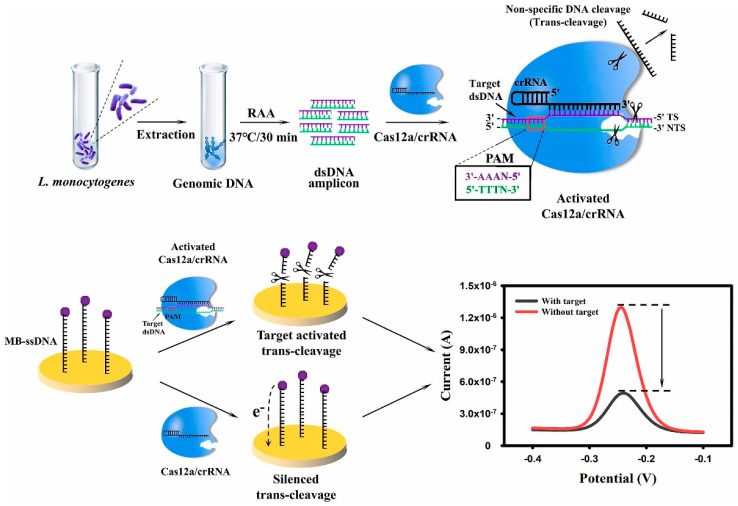
RAA-based E-CRISPR biosensor for *Listeria monocytogenes* detection.

Li et al. [113] proposed a label-free *listeria monocytogenes* detection method using CRISPR-Cas12a combined with an electrochemical transduction strategy. As an electrochemical probe, methylene blue (MB) can be reduced by the transfer of two electrons, and one proton produces an electrochemical signal (Figure 9). The researchers immobilized the MB-ssDNA reporter at the electrode. In the presence of the target bacteria, the trans-cleavage ability of Cas12a is activated by the RAA-amplified target nucleic acid, cleaving the MB-ssDNA reporter off the electrode surface, resulting in a decrease in the number of electrons transferred and a decrease in the current. When the target is not present, the current remains unchanged. Under optimized conditions, RAA-based E-CRISPR can detect as low as 0.68 aM of genomic DNA and 26 cfu/mL of *L. monocytogenes* in pure cultures. In addition, some new conductive materials have lower resistance and larger contact areas, which can be used to improve the performance of electrochemical detection, such as the use of AuNP and MXene to modify the probe and electrode surfaces, respectively, which can effectively amplify the electrochemical signal and improve the detection sensitivity [114,115].

Electrochemical signal detection has the advantage of low cost and portability compared to fluorescence detection. A non-specific ssDNA reporter modified with methylene blue (MB) at the 3′ end, which acts as a redox indicator and generates current change for signal transduction [116], and at the 5′ end with a sulfhydryl fragment, which is immobilized on a gold nanomaterial-modified electrode via an Au-S bond. Thus, the process of electron transfer between the redox species on the gold electrode and ssDNA can be initiated and transduced by electrochemistry. In the presence of a target, Cas12a trans-cleavage activity is activated, and the MB-ssDNA reporter is cleaved from the electrode surface, thereby reducing MB signaling [117]. Zheng et al. [72] reported for the first time an electrochemical biosensor based on saltatory rolling circle amplification (SRCA) combined with a CRISPR-Cas12a system for the detection of *Salmonella*. The detection range of this method was 5.8 fg/μL~5.8 ng/μL, and the LOD was 2.08 fg/μL. The biosensor showed good sensitivity, accuracy, and specificity for the detection of *Salmonella* in real samples, which was consistent with real-time fluorescence quantitative PCR (RT-qPCR). The biosensor provides an effective platform for the detection of *Salmonella* in food and provides technical support for the detection of other foodborne pathogens, potentially being used to detect other foodborne pathogens.

Bonini et al. [73] established a label-free impedance biosensing assay coupled with electrochemical impedance spectroscopy (EIS) measurements for the detection of *Escherichia coli* and *Staphylococcus aureus*. EIS is a sensitive and powerful technique detecting subtle physical and chemical changes occurring at the electrode surface. ssDNA immobilized on the electrode can hinder the electron exchange between the electrode and the solution. When Cas12a/gRNA system recognizes a specific sequence of amplicons from different clinical isolates of *E. coli* and *S. aureus*, it activates cleavage activity on the ssDNA, leading to a decrease in charge transfer resistance. Bonini et al. propose an easy-use, rapid, and low-cost detection system based on a label-free ssDNA immobilized on a gold electrode with a limit of detection of 3 nM and a short turnaround time of approximately 1.5 h.

### 3.4. Other Biosensor Assays Based on the CRISPR/Cas System

In addition to fluorescence, colorimetric, and electrochemical signal output, the CRISPR/Cas12 system can also convert and output the signal of biomolecules into surface-enhanced Raman spectroscopy (SERS) signals, which can be used for quantitative detection on the basis of qualitative analysis. Liu et al. [118] proposed a SERS biosensor mediated by CRISPR/Cas12a strategy for on-site detection of nucleic acids. The target DNA was amplified using LAMP, and the ssDNA was modified on the electrode with a liposome-encapsulated signaling molecule at one end. Cas12a recognizes the target DNA and uses trans cleavage activity to cleave the ssDNA on the electrode, resulting in a decrease in ssDNA concentration. After the addition of surfactant, the liposome ruptures and releases signaling molecules, which can be used for the detection of SERS signals. The method has been successfully applied to the adulteration analysis of duck meat, with a detection limit as low as 10 pmol/L. RPA-assisted CRISPR/Cas12a detection of SERS signals on microfluidic paper-based analytical devices (RPA-Cas12a-μPAD) enables detection of *Salmonella typhimurium* as low as 3–4 CFU/mL in milk and meat samples [42].

Zhuang et al. [42] developed an integrated paper-based microfluidic device for the detection of *Salmonella typhimurium* by combining the advantages of SERS and CRISPR-Cas12a. The thiolated ssDNA conjugated gold nanostar@4-mercaptobenzoic acid@goldnanoshell structures (AuNS@4-MBA@Au@DNA) were prepared in which the 4-mercaptobenzoic acid were used as signal molecules for SERS detection. When the target is present, the trans-cleavage of CRISPR/Cas12a was activated, resulting in the indiscriminate shredding of linker ssDNA. The limit of detection for *S. typhi* was approximately 3–4 CFU/mL for spiked milk and meat samples within 45 min. In addition, the paper-based detection method has the advantages of simple fabrication, low cost, and easy use to meet the needs of POCT [119], while CRISPR-Cas12a and SERS also make up for the shortcomings of the low sensitivity of paper-based detection, and with the rapid development of handheld Raman spectrometers and small Raman spectrometers, this method will be more suitable for POCT platforms.

Microfluidic devices integrate experimental operations such as sample preparation, reaction, separation, and detection and analysis into a micron-sized chip, which is usually composed of a variety of materials such as glass, quartz, polymer materials, hydrogels, and paper, and has the characteristics of automation, miniaturization, high throughput and low cost [120]. Compared with other microfluidic devices, paper-based microfluidic devices (μPADs) have many advantages: a. low cost of paper, which is a renewable resource; b. without external force, and the sample can be driven for analysis by capillary force; c. good flexibility; d. Pre-storage of reaction reagents, etc. [119], which can be a powerful tool for the detection of foodborne pathogens [77,78,121]. Despite the above advantages, μPADs still have the disadvantages of low detection sensitivity and limited quantitative ability, which may be due to changes in reagent activity stored on μPADs, uneven mixing of reagents, and inappropriate concentration [122,123].

**Figure 10:**
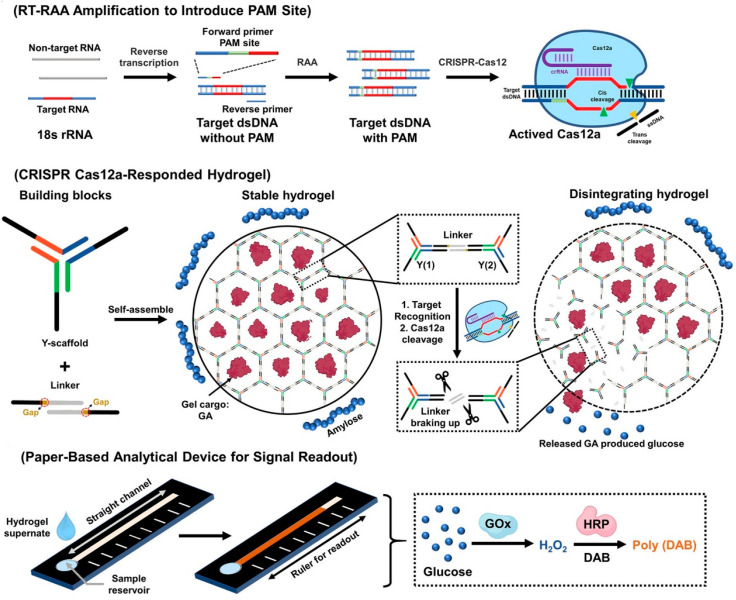
Microfluidic ruler-readout and CRISPR Cas12a-responded hydrogel-integrated paper-based analytical devices (μReaCH-PAD) for visible testing.

In recent years, researchers have explored various techniques to promote the design and modification of μPADs; for example, Huang et al. [124] developed a μPAD that integrates the CRISPR/Cas12a system and hydrogel for quantitative detection of fungal (Figure 10). The hydrogel consisted of Y-shaped scaffold DNA and linker DNA by self-assembly and retained ssDNA ligation sites for cleavage of the Cas12a protein. The authors first reverse transcribed the 18s rRNA of the fungus to obtain dsDNA with the PAM site, the cleavage activity of Cas12a protein can be activated by dsDNA, and the hydrogel disintegrates and releases glucoamylase. At this time, the straight-chain starch in the solution is hydrolyzed to produce a large amount of glucose. Glucose oxidase pre-loaded in the sampling wells of μPADs can oxidize the glucose in the solution to produce H_2_O_2_. H_2_O_2_ is driven by capillary forces and employed by horseradish peroxidase preinstalled in the reaction channel to convert diaminobenzidine (DAB) to brown DAB. The concentration of glucose in the μPADs is positively correlated with the length of the brown band, allowing for the estimation of fungal content based on the length of the brown band. The detection limit of this device for several bacteria can be as low as 10 CFU/mL by the naked eye, which realizes the quantitative detection of the target.

**Figure 11:**
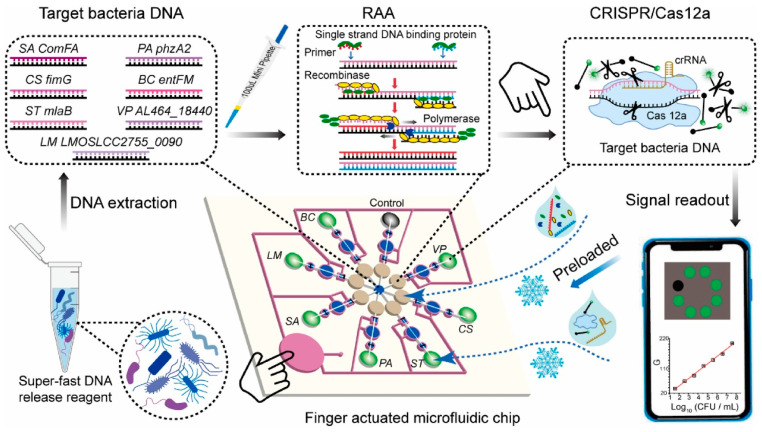
Multiplexed detection of foodborne pathogens using one-pot CRISPR/Cas12a on a finger-actuated microfluidic biosensor.

In addition to paper-based microfluidics, other types of microfluidic chips based on CRISPR/Cas systems have been investigated for the detection of foodborne pathogens, such as finger-actuated microfluidic chips combining RAA and one-pot CRISPR/Cas12a for the simultaneous detection of a variety of foodborne pathogens [74], microfluidic chips combining the CRISPR/Cas13a system for the detection of *Listeria monocytogenes* [57], and digital microfluidics based on the CRISPR/Cas12a system for detecting *Staphylococcus aureus* [75] (Figure 11). Overall, the development of microfluidic detection methods for foodborne pathogens based on the CRISPR/Cas system has great advantages and prospects for on-site detection.

**Figure 12:**
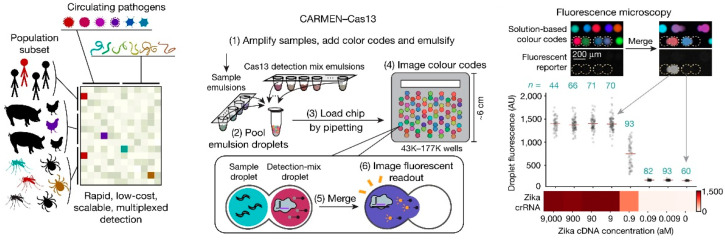
CARMEN platform for multiplexed pathogen detection.

At present, CRISPR/Cas is widely used for the detection of single-species pathogens, but contamination of multiple-species pathogens often exists in food, and single-species pathogen detection methods are difficult to meet the demand for simultaneous analysis of multiple pathogens. The detection of mixed contaminated pathogenic bacteria in food through the multiplex detection strategy can effectively improve detection efficiency and save labor and reagent costs. Since the trans-cleavage activity of Cas12 and Cas13a is not selective for nucleic acid sequences, researchers used a single Cas protein combined with techniques such as microfluidics, machine learning, and spatial coding to distinguish different signals. Qian et al. developed a slip valve-assisted microfluidic chip coupling with CRISPR/Cas12a for dual nucleic acid analysis. The designed Cas12a detection reagents were preloaded on the fluidic chip, the duplex LAMP amplicons were mixed with two CRISPR detection units controlled by slip valves and syringes, and the results could be observed by the naked eye under a portable ultraviolet lamp. The method can detect as low as 30 copies/reaction of *V. parahaemolyticus* and 20 copies/reaction of *S. typhimurium*. Bruch et al. [125] developed a CRISPR/Cas13a-mediated microfluidic multiplex electrochemical sensor. In the presence of the target RNA, the trans-cleavage activity of the Cas13a/crRNA is activated, and the reporter RNA strand is cleaved. Multiple detection is realized by designing multiple channels on microfluidics chips to distinguish different targets. This method can be used to detect miR-19b and miR-20a with a detection limit of 10 nmol/L. In order to improve the detection capability based on CRISPR, the combinatorial arrayed reactions for multiplexed evaluation of nucleic acids (CARMEN) detection platform were developed by combining CRISPR diagnosis and microfluidic technology. Ackerman et al. [126] developed the CARMEN platform for multiplexed pathogen detection (Figure 12). In the CARMEN platform, nanolitre droplets containing CRISPR-based nucleic acid detection reagents are dispersed in a microarray. When the multiplexed nucleic acid amplification products are added to the microarray, the CRISPR reagents pair with droplets of amplification products, causing Cas13a to cleave the reporter RNA and generate a fluorescent signal. The combination of CARMEN and Cas13 detection (CARMEN-Cas13) enables a multiplexed assay that simultaneously differentiates all 169 human-associated viruses, as well as comprehensively subtyping influenza A strains. In addition, a multiplexed detection system termed Cas-Loaded Annotated Micro-Particles (CLAMP) can also be constructed using spatially encoded hydrogel microparticles and machine learning techniques [127]. The approach compartmentalizes the CRISPR/Cas reaction in spatially encoded hydrogel microparticles (HMPs). Each HMP is identifiable by its face code and becomes fluorescent when target DNA is present. The CLAMP assay is fast, highly sensitive (attomolar detection limits with preamplification), and capable of multiplexing in a single-pot assay.

The CRISPR-Cas system can also be used to detect non-nucleic acid targets, and the non-nucleic acid signals are converted to nucleic acid signals through signal transduction generated by the allosteric modulation of functional aptamers [128]. Shen et al. [60] combined allosteric nucleic acid probes with the Cas13a system to detect *Salmonella* enteritidis with the same or even higher sensitivity than RT-PCR. The probe is divided into three parts, including an aptamer that recognizes the bacterial antigen, primer binding site and T7 promoter binding site. In the absence of the target bacteria, the allosteric probe maintains the hairpin structure and inactive configuration, which is unable to bind to the primers to initiate transcription. In the presence of the target bacteria, the allosteric probe binds to the target bacteria, resulting in the opening of the hairpin structure. The DNA polymerase binds to the T7 RNA polymerase to initiate transcription, and the Cas13a enzyme recognizes the RNA transcript and cleaves the reporter molecule to produce a fluorescent effect.

## 4. Summary and Outlook

CRISPR/Cas-based biosensors have good generality and programmability for detecting a wide range of foodborne pathogens. With intelligent design, many CRISPR/Cas-coupled nucleic acid amplification methods with high sensitivity can detect target nucleic acids as low as amol/L. Combining CRISPR/Cas with signal output modes such as SERS, lateral flow immunochromatography, and chromogenic and fluorescent methods allows for the detection of foodborne pathogens without the use of specialized instrumentation and facilitates on-site testing.

However, there are still some challenges with CRISPR/Cas-based detection technologies. (1) CRISPR/Cas biosensor is still at the stage of single foodborne pathogen detection mode, and the requirement of PAM or PFS sequence for Cas effectors proteins limits the detection of certain sequences [129]. Realizing multiple detection under one-pot is crucial for food safety; although detection methods without PAM sequences have been developed, the sensitivity and specificity of these methods are poor, and it is necessary to further study the mechanism of action of Cas proteins, obtain some artificially modified Cas proteins, and increase the types of Cas proteins or the selectivity of their PAM sequences, so as to enable their use in the non-traditional PAM sequence-dependent nucleic acid sequence detection, to realize the simplicity of the multiple detection process. (2) Most CRISPR/Cas-based biosensors still suffer from complex working procedures involving nucleic acid extraction and nucleic acid amplification. The sample pretreatment process of nucleic acid cannot be overlooked, which may cause false-positive results, increase the risk of cross-contamination, and is time-consuming. (3) CRISPR/Cas-based rapid detection technology cannot distinguish between live and dead bacteria, which can result in false-positive outcomes. To address this drawback, aptamers can be employed to differentiate between live and dead bacteria. Nevertheless, this approach is relatively complex, and a more effective method needs to be explored in future research. (4) Most of the CRISPR/Cas-based sensor systems were operated according to nucleic acid determination. Hence, the developing non-nucleic acid target detection remains to be further explored. Elaborately, a few biosensors coupled with CRISPR/Cas system are designed for the quantification of non-nucleic-acid targets like metal ions, proteins, and adenosine triphosphate (ATP). There are still several obstacles to developing more non-nucleic acid target platforms that integrate CRISPR systems with biological receptors, such as antibodies, aptamers, and enzymes [130,131]. (5) In the process of on-site detection, complex sample matrices may affect the detection effect, and it is necessary to pretreat the test samples such as extraction and purification, so the development of simple and effective sample extraction devices is also an important means to improve the detection efficiency. In addition, in the process of constructing analytical methods based on the CRISPR/Cas system, most of them involve three main steps of nucleic acid extraction, amplification, and detection, and if these three steps can be integrated into the same LFAs test strip or μPADs, the simplicity and detection efficiency will be greatly improved. (6) Although optimized crRNA can reduce off-target effects in CRISPR/Cas assays, off-target phenomena are difficult to avoid in actual assays and may lead to misclassification of results. Therefore, enhancing the evaluation of off-target effects by computer algorithms and the design of crRNA sequences to improve the target recognition of CRISPR/Cas will be an important research direction. (7) Establish an AI-based CRISPR system for the analysis and interpretation of CRISPR test results to further improve detection performance and eliminate any subjective results associated with the operator’s interpretation. A CRISPR-based intelligent diagnostic device was developed that integrates automatic image acquisition and algorithm processing to further reduce the analysis time and subjectivity of CRISPR assays and to produce digitized results. (8) The non-specific trans-cleavage activity of Cas proteins makes it difficult to distinguish signals of different targets in multiplexed assays, and multiplexed assays still face great challenges. The development and promotion of microfluidic chips and the reduction in chip and instrument costs will greatly promote the popularization and application of multiplex detection methods. (9) Currently, the combination of nucleic acid amplification and CRISPR/Cas can only qualitatively analyze pathogenic bacteria, and the quantitative detection performance still needs to be improved. Exploring the kinetic mechanism of nucleic acid amplification and CRISPR/Cas cutting reaction process, studying the interaction mechanism between nucleic acid amplification and CRISPR/Cas cutting reaction, and reducing the mutual inhibition between CRISPR/Cas and nucleic acid amplification are important ways to realize quantitative detection. (10) Some of the above detection methods have not yet been expanded and applied to the detection of foodborne pathogens, but they are useful for the development of foodborne pathogens sensors.

In summary, the combination of CRISPR/Cas detection technology with colorimetric biosensors, fluorescent biosensors, and electrochemical biosensors shows great promise in the detection of food pathogens. However, these approaches are still in their infancy and there are still some problems to be solved. In addition, the standards and technical specifications for the CRISPR/Cas sensors still need to be greatly improved. With the development and progress of various related technologies, CRISPR/Cas-based biosensors will play an increasingly important role in the on-site detection of foodborne pathogens.

## Figures and Tables

**Table 1:** Structural characteristics and functions of the Class 2 CRISPR-Cas system.

Effector Proteins	Cas9	Cas12a	Cas12b	Cas13a	Cas14a
Molecular size	1000–1400 aa	1100–1300 aa	1100–1300 aa	900–1000 aa	400–700 aa
Type	type II	type V	type V	Type VI	type V
Domain	HNH RuvC	RuvC Nuc	RuvC	HENP	RuvC
Guide RNA	tracrRNA crRNA	crRNA	tracrRNA crRNA	crRNA	tracrRNA crRNA
PAM/PFS	5′-NGG	5′-TTTN	5′-TTN	PFS 3′ non-G	Not for ssDNA, 5′ T-rich PAM for dsDNA
Target	dsDNA	(ds/ss)DNA	(ds/ss)DNA	ssRNA	ssDNA
Cis-cleavage	blunt	5′ staggered cut	5′ staggered cut	Near U or A	staggered
Trans-cleavage	no	specific ssDNA	specific ssDNA	specific ssRNA	specific ssDNA
Sensitivity	Attomolar	Attomolar	Attomolar	Attomolar	Attomolar
Specificity	single-base mismatch distinction	single-base mismatch distinction	single-base mismatch distinction	single-base mismatch distinction	single-base mismatch distinction

**Table 2:** CRISPR/Cas technology-based application platform for foodborne pathogen testing.

Effector Proteins	Detection of Targets	Signal Output	Signal Amplification	Sensitivity	Detection Time	References
Cas9	*E. coli O157:H7*	Fluorescence	SDA and RCA	40 CFU/mL	120 min	[34]
Cas9nAR	*Salmonella typhimurium* and *Escherichia coli*	Colorimetric	SDA	100 copies, 100 CFU/mL	~3 h	[35]
Cas9 nAR	*Salmonella typhimurium*, *E. coli O157:H7*	fluorescence Colorimetric	LAMP	5.8 copies	60 min	[36]
(d)Cas9	African Swine Fever, *Listeria* *monocytogenes*, GMOs	Colorimetric/LFA	PCR/RPA	100 copies	1 h	[37]
SpCas9	methylation and *L. monocytogenes*	Fluorescence	EXPAR	0.82 aM	<1 h	[38]
Cas9	*L. monocytogenes*	Colorimetric	EXPAR	100 aM	-	[39]
dCas9	*MRSA bacterium*	Fluorescence		10 CFU/mL	<30 min	[40]
Cas9, Cas12, Cas13	ASFV, *Staphylococcus* *aureus*, *Listeria* *monocytogenes*, *P. aeruginosa*	Fluorescence/ Colorimetric	RPA	150 copies	-	[41]
Cas9	*Salmonella Typhimurium*	Colorimetric/LFSs	PCR	100 CFU/mL	-	[42]
dCas9	*Visceral leishmaniasis*	colorimetric	RCA	10 copies	15 min	[43]
Cas12a	*S. typhimurium*	SERS signals	RPA	3–4 CFU/mL	45 min	[44]
Cas12a	*V. parahaemolyticus*	Fluorescence	LAMP	1.36 × 10^2^ copies	-	[45]
Cas12a	*Escherichia coli*	Fluorescence	RPA	10 copies	45 min	[46]
Cas13a	*Salmonella* spp.	-	RPA	100 copies	20 min 45 min	[47]
Cas12a	*E. coli O157:H7*	Fluorescence	RAA	1 CFU/mL	55 min	[48]
Cas12a	*L. monocytogenes*	Fluorescence	RPA	10 CFU/mL	50 min	[49]
Cas12a	*L. monocytogenes*	Fluorescence	RAA	4.4 CFU/g	25 min	[50]
Cas12a	*Staphylococcus* *aureus*	Colorimetric	RAA	75 aM	70 min	[51]
Cas12a	*Vibrio* *parahaemolyticus*	Fluorescence	RAA	67 CFU/mL	60 min	[52]
Cas12a	*Vibrio parahaemolyticus*	Fluorescence	LAMP	30 copies/ reaction	50 min	[53]
Cas12a	*E. coli O157:H7*	Fluorescence	LAMP	1.22 CFU/mL	70 min	[54]
Cas12a	*Vibrio parahaemolyticus*	Fluorescence	LAMP	2.5 CFU/mL	25 min	[55]
Cas12a	*Shigella flexneri*	Colorimetric	LAMP	4 copies/μ	40 min	[56]
Cas12a	*Salmonella*	Fluorescence	free	84 CFU/mL	30 min	[57]
Cas12b	*C. jejuni*	Fluorescence	-	10 CFU/g	40 min	[58]
Cas13a	*Listeria*	Fluorescence	RAA	0.32 aM	60 min	[59]
Cas14a	*S. aureus*	Fluorescence	-	400 CFU/mL	150 min	[11]
Cas12a	*E. coli O157:H7* and *Streptococcus aureus*	Fluorescence	RPA	1 CFU/mL	50 min	[60]
LwaCas13a	*E. coli*, *K.* *pneumoniae*; *P. aeruginosa*; *Mycobacterium* *tuberculosis*; *Staphylococcus* *aureus*.	Fluorescence	RPA RT-RPA	aM	2–5 h	[61]
LwaCas13a	*Salmonella* Enteritidis	Fluorescence	Isothermal amplification +T7 transcription	1 CFU/mL	<2 h	[62]
Cas12a	*MRSA*	Colorimetric	RPA	8 CFU/mL	-	[63]
Cas12a	*Salmonella*	Colorimetric	G-quadruplex	1 CFU/mL	-	[64]
Cas12a	*V. parahaemolyticus*	Colorimetric	LAMP	6.1 × 10^2^ CFU/mL	-	[65]
Cas12a	*MRSA*	Colorimetric	RPA	0.2 aM	2 h	[66]
Cas12a	*Staphylococcus aureus*	Colorimetric	LFA	20 CFU/mL	1 h	[67]
Cas12a	*S. aureus*	Fluorescence Colorimetric	RPA	5 copies	35 min	[68]
Cas12a	*MRSA*	electrochemical	free	10 fM	-	[69]
Cas12a	*S. aureus*	electrochemical	SRCA	3 CFU/mL	-	[70]
Cas12a	*E. coli O157:H7*	electrochemical	RCA	10 CFU/mL	-	[71]
LbCpf1	*S. aureus*	electrochemical	SDA	0.437 fM	-	[72]
Cas12a	*E. coli O157:H7*	electrochemical	PER	19 CFU/mL	-	[73]
Cas12a	*Salmonella*	electrochemical	SRCA	2.08 fg/μL	-	[42]
Cas12a	*E. coli* and *S. aureus*	electrochemical	free	3 nM	1.5 h	[74]
Cas12a	*Salmonella typhimurium*	SERS	RPA	3–4 CFU/mL	45 min	[75]
Cas12a	*multiplex*	Colorimetric	RAA	500 CFU/mL	1 h	[76]
Cas12a	*Staphylococcus aureus*	Fluorescence	RPA	32 CFU/mL	55 min	[77]
Cas12a	*V. parahaemolyticus*, *S. typhimurium*	Colorimetric	LAMP	30 copies 20 copies	60 min	[78]

## Data Availability

Data are contained within the article.
